# Supplementary material for: Community engagement in health services research on soil-transmitted helminthiasis in Asia Pacific region: Systematic review
Source: PLOS Glob Public Health. 2023 Mar 22;3(3):e0001694. doi: 10.1371/journal.pgph.0001694 (PMC10032488; doi:10.1371/journal.pgph.0001694)
Supplement: S1 Table — (DOC) [file pgph.0001694.s002.doc]

**S1 Table.** **Search strategy and Information sources**

**Ovid Full text**

| # | Query | Results |
| --- | --- | --- |
| 1 | community.mp. [mp=title, abstract, full text, caption text] | 144,458 |
| 2 | community engagement.mp. [mp=title, abstract, full text, caption text] | 1,160 |
| 3 | participatory.mp. [mp=title, abstract, full text, caption text] | 2,727 |
| 4 | action research.mp. [mp=title, abstract, full text, caption text] | 1,264 |
| 5 | participatory research.mp. [mp=title, abstract, full text, caption text] | 823 |
| 6 | participatory action research.mp. [mp=title, abstract, full text, caption text] | 237 |
| 7 | community-based research.mp. [mp=title, abstract, full text, caption text] | 383 |
| 8 | action science.mp. [mp=title, abstract, full text, caption text] | 26 |
| 9 | action inquiry.mp. [mp=title, abstract, full text, caption text] | 9 |
| 10 | cooperative inquiry.mp. [mp=title, abstract, full text, caption text] | 11 |
| 11 | health service research.mp. [mp=title, abstract, full text, caption text] | 423 |
| 12 | helminth$.mp. [mp=title, abstract, full text, caption text] | 986 |
| 13 | 1 or 2 or 3 or 4 or 5 or 6 or 7 or 8 or 9 or 10 | 145,715 |
| 14 | ascaris$.mp. [mp=title, abstract, full text, caption text] | 165 |
| 15 | hookworm$.mp. [mp=title, abstract, full text, caption text] | 135 |
| 16 | trichuris$.mp. [mp=title, abstract, full text, caption text] | 101 |
| 17 | 12 or 14 or 15 or 16 | 489 |
| 18 | 11 and 17 | 238 |

| Source | hits |
| --- | --- |
| Ovid full text | 238 |
| PubMed central | 99 |
| Scopus | -Lancet general health (12)  -Lancet public health (0)  -BMC public health (40 found)  None selected. |
| Best Evidence Medical Education | No relevant studies |
| PsycInfo | Not accessible |
| Database of Abstracts of Reviews of Effectiveness | No relevant studies |
| Regional bibliographic databases   1. Australia Australian Education Index (www.acer.edu.au/library/ aei/index.html) | No relevant studies |
| 1. Britain British Education Index (www.leeds.ac.uk/bei/index. html) | 5 |
| 1. Canada CBCA Education (www.twu.ca/Library/cbcaeduc.htm) | No relevant studies |
| System for Information on Grey Literature | No relevant studies |
| Dissertations and theses databases   1. ProQuest (dissertation databases) | 13 |
| 1. Theses Canada (Library and Archives Canada) | No relevant studies |
| 1. Networked Digital Library of Theses and Dissertations | 27 |
| Full‐text journals available electronically   1. Directory of Open Access Journals (DOAJ) | No relevant studies |
| 1. Education Research Global Observatory | Not accessible |
| Google Scholar | 3080 |
| CENTRAL & Cochrane Collaboration Library | 4 (trials) |
| Research Registers and Websites | No relevant studies |
| Science direct | 5 |
