# Supplementary material for: Community engagement in health services research on soil-transmitted helminthiasis in Asia Pacific region: Systematic review
Source: PLOS Glob Public Health. 2023 Mar 22;3(3):e0001694. doi: 10.1371/journal.pgph.0001694 (PMC10032488; doi:10.1371/journal.pgph.0001694)
Supplement: S2 Table — (DOC) [file pgph.0001694.s003.doc]

S2 Table. Excluded studies and reasons for exclusion

| No. | Study, yr | Reason for exclusion | Citation |
| --- | --- | --- | --- |
| 1 | Dreyfuss, 2000 | Not about CE | Dreyfuss M L, Stoltzfus R J, Jaya B S, et al. Hookworms, malaria and vitamin A deficiency contribute to anaemia and iron deficiency among pregnant women in the plains of Nepal. Journal of Nutrition 2000: 55, 2527–2536 |
| 2 | Wani, 2008 | Not about CE | Wani, S., Ahmad, F., Zargar, S. et al (2008). Intestinal helminths in a population of children from the Kashmir valley India; J of Haematology 82(4) 313-317 |
| 3 | Massa, 2009 | Not in the Asia Pacific region | Massa K, Magnussen P, Sheshe A, et al. The effect of the community-directed treatment approach versus the school-based treatment approach on the prevalence and intensity of schistosomiasis and soil-transmitted helminthiasis among schoolchildren in Tanzania. Transactions of the Royal Society of Tropical Medicine and Hygiene,2009 103, 31-37. |
| 4 | Nath, 2018 | Knowledge gap assessment | Nath TC, Padmawati RS, Murhandarwati EH. Barriers and gaps in utilization and coverage of mass drug administration program against soil-transmitted helminth infection in Bangladesh: Implementation research. J Infect Public Health. 2019 Mar-Apr;12(2):205-212. |
| 5 | Salam, 2017 | Prevalence study | Salam N, Azam S. Prevalence and distribution of soil-transmitted helminth infections in India. *BMC Public Health* **17**, 201 (2017). |
| 6 | Salam, 2014 | Systematic review | Salam RA, Haider BA, Humayun Q, et al. Effect of administration of antihelminthics for soil-transmitted helminths during pregnancy. Cochrane Database of Systematic Reviews 2015, Issue 6. Art. No.: CD005547. DOI: 10.1002/14651858.CD005547.pub3. |
| 7 | Taylor-Robinson, 2019 | Systematic review | Taylor-Robinson DC, Maayan N, Soares-Weiser K, et al. Deworming drugs for soil-transmitted intestinal worms in children: effects on nutritional indicators, haemoglobin and school performance. Cochrane Database Syst Rev. 2012 Nov 14;11:CD000371 |
| 8 | Parikh, 2013 | KAP study | Parikh DS, Totanes FI, Tuliao AH, et al. Knowledge, attitudes and practices among parents and teachers about soil-transmitted helminthiasis control programs for school children in Guimaras, Philippines. Southeast Asian J Trop Med Public Health. 2013 Sep;44(5):744-52 |
| 9 | Clarke, 2016 | Protocol only | Clarke, N.E., Clements, A.C.A., Bryan, S. *et al.* Investigating the differential impact of school and community-based integrated control programmes for soil-transmitted helminths in Timor-Leste: the (S)WASH-D for Worms pilot study protocol. *Pilot Feasibility Stud* 2016: 2, 69. |
| 10 | Clarke, 2019 | Drug efficacy study | Clarke NE, Doi SAR, Wangdi K, et al. Efficacy of Anthelminthic Drugs and Drug Combinations Against Soil-transmitted Helminths: A Systematic Review and Network Meta-analysis, Clinical Infectious Diseases, 2019; 68:96–105, |
| 11 | Anderson, 2013 | Secondary data analysis, not on CE | Anderson RM, Truscott JE, Pullan RL, Brooker SJ, Hollingsworth TD. How effective is school-based deworming for the community-wide control of soil-transmitted helminths? PLoS Negl Trop Dis. 2013;7(2):e2027 |
| 12 | Sanza, 2011 | Trend assessment, not about CE | Sanza M, Totanes FI, Chua PL, et al. Monitoring the impact of a mebendazole mass drug administration initiative for soil-transmitted helminthiasis (STH) control in the Western Visayas Region of the Philippines from 2007 through 2011. Acta Trop. 2013;127(2):112-7. |
| 13 | Belizario, 2014 | not about CE | Vicente Belizario, Jr, Paul Lester Chua, Harvy Joy Liwanag, et al. Soil-Transmitted helminthiases in secondary school students in selected sites in two provinces in the Philippines: Policy Implications, Journal of Tropical Pediatrics, 2014; 60 (4):303–7. |
| 14 | Mationg, 2017 | not about CE | Mationg MLS, Gordon CA, Tallo VL, et al. Status of soil-transmitted helminth infections in schoolchildren in Laguna Province, the Philippines: Determined by parasitological and molecular diagnostic techniques. PLoS Negl Trop Dis.2017; 11(11): e0006022. H |
| 15 | Income, 2021 | not about CE | Income N, Tongshoob J, Taksinoros S, et al. Helminth Infections in Cattle and Goats in Kanchanaburi, Thailand, with Focus on Strongyle Nematode Infections. Vet Sci. 2021 ;8(12):324. |
| 16 | Ngui, 2011 | not about CE | Ngui R, Ishak S, Chuen CS, et al. Prevalence and risk factors of intestinal parasitism in rural and remote West Malaysia. PLoS Negl Trop Dis. 2011;5(3):e974. |
| 17 | Ngui, 2015 [ | not about CE | Ngui R, Aziz S, Chua KH, et al. Patterns and risk factors of soil-transmitted helminthiasis among Orang Asli subgroups in Peninsular Malaysia. Am J Trop Med Hyg. 2015;93:361-70. |
| 18 | Sjafii, 2020 | ethical consideration-related action | Sjafii RP, Ramayani OR, Pasaribu S, et al. The effectiveness of school-based and community-based deworming methods in primary school children with soil-transmitted helminth infection. Open Access Maced J Med Sci. 2020;8(E):296-301. |
| 19 | Al-Mekhlafi, 2007 | ethical consideration-related action | Al-Mekhlafi MS, Atiya AS, Lim YA, et al. An unceasing problem: soil-transmitted helminthiases in rural Malaysian communities. Southeast Asian J Trop Med Public Health. 2007; 38:998-1007. |
| 20 | Ahmed, 2012 | ethical consideration-related action | Ahmed A, Al-Mekhlafi HM, Azam MN, et al. Soil-transmitted helminthiasis: a critical but neglected factor influencing school participation of Aboriginal children in rural Malaysia. Parasitology. 2012;139:802-8. ` |
| 21 | Bait, 2019 | Not STH | Bait BR, Rah JH, Roshita A, et al. Community engagement to manage acute malnutrition: implementation research in Kupang district, Indonesia. Bull World Health Organ. 2019 Sep 1;97(9):597-604. |
| 22 | Mationg,2021 | not about CE | Mationg MLS, Williams GM, Tallo VL, et al. "The Magic Glasses Philippines": a cluster randomised controlled trial of a health education package for the prevention of intestinal worm infections in schoolchildren. Lancet Reg Health West Pac. 2021;18:100312. |
| 23 | Vaz Nery,2019 | Not about CE | Vaz Nery S, Traub RJ, McCarthy JS, et al. WASH for WORMS: A Cluster-Randomized Controlled Trial of the Impact of a Community Integrated Water, Sanitation, and Hygiene and Deworming Intervention on Soil-Transmitted Helminth Infections. Am J Trop Med Hyg. 2019;100(3):750-761. |
| 24 | Ramil,2019 | Not STH | ​Ramli NAB, Rahman NAB, Ali HAM, et al. Pre-school young doctor (tunas doktor muda) programme and the development of good health behaviour among pre-school children in Pahang. International Journal for Studies on Children, Women, Elderly And Disabled. 2019 |

CE: Community engagement; KAP: knowledge, attitude and practice
