# Supplementary material for: Community engagement in health services research on soil-transmitted helminthiasis in Asia Pacific region: Systematic review
Source: PLOS Glob Public Health. 2023 Mar 22;3(3):e0001694. doi: 10.1371/journal.pgph.0001694 (PMC10032488; doi:10.1371/journal.pgph.0001694)
Supplement: Attachment — Submitted filename: Our reply _ comments _ 27 Dec.pdf [file pgph.0001694.s004.pdf]

Our reply to the comments

PGPH-D-22-01639

Community Engagement in Health Services Research on Soil-Transmitted Helminthiasis in Asia Pacific Region: Systematic review

Journal Requirements:

Additional Editor Comments (if provided):

Thank you for submitting your article. Both peer reviewers have questioned aspects of the search methodology, including the choice/variety of key words as well as the exclusion criteria, in part as the current approach seems to have excluded several articles which appear to address the topic of community engagement in STH programs in the Asia-Pacific Region. Please ensure that these comments are robustly addressed, either through changes to the methodology or additional explanation and justification in the manuscript, when submitting a revised manuscript. Please also note the additional minor comments made by each reviewer.

Our reply/

We have updated the keywords and the search strategy, accordingly.

Sincerely, we believe our inclusion and exclusion criteria are thorough. A study should be included if programme/intervention address community/stakeholder involvement or engagement and provide mechanisms and/or processes of community engagement. Moreover, it should be targeted towards health services context. Thank you.

Reviewer #1: General: The review focuses on an important and increasingly prominent topic, namely the involvement of study communities in scientific studies, here those conducted in South-East Asia and dealing with soil-transmitted helminths. Only 8 relevant publications were identified. The manuscript is easy to follow and generally well written. This reviewer

has not fundamental concerns about the study but offers a number of comments to further improve the manuscript:

Q/

- Line 78: “people and the act that is the most common cluster” – the second part of the sentence is unclear. Consider revising.

Our reply/

For clarity, we have rephased this sentence. Thank you

In the revised version

Line # 81-83

Of these, soil-transmitted helminth infection (STH) represents the most prevalent cluster of NTDs worldwide [5] and receives less attention than would be suggested by the burden it takes on people.

Q/

- Line 131: why was “trichuriasis” not included as keyword when “hook worms” and “ascariasis” were?

Our reply/

In the revised version

Line # 132-135

The keywords with appropriate Bolen operators were used: “community engagement” “community participatory” “action research” “participatory research” “participatory action research” “community-based research” “soil transmitted helminths" " hook worms" " ascariasis" “ trichuriasis”.

Q/

- Line 141: “Asian” should be “Asia”

Our reply/

We have corrected it. Thank you

In the revised version

Line #144-145

Communities residing in LMICs in WHO defined Western Pacific and South East Asia, regardless of age and gender. Communities is as defined in the primary studies.

Q/- Line 203: "0+ 1+ 0+0++1= 2)" should be "0+ 1+ 0+0+1= 2)"

Our reply/

We have corrected it. Thank you

In the revised version

Line #204-207

As an example, study X showed CE as 'consultation' at 'developing ideas', collaboration at 'developing methodology', and 'informed' at data collection/analysis, but 'no information' at reporting writing and 'collaboration' at dissemination stage,) achieved a total score of 2 (i.e., 0+ 1+ 0+0+1= 2).

Q/- Table 1 and Table 3: check format – the chosen format is unsuitable for publication due to very long and narrow columns.

Our reply/

We have reformatted Table 1 and Table 2. Thank you

Q/

- Lines 309-315: it is unclear why this paragraph appears here as it does not contribute to the topic of interest: it focuses on barriers to MDA acceptance, not community involvement

Our reply/

This paragraph has been improved for clarity in this revised version. The majority of studies attributed community involvement with community-based mass drug administration. As a result, barriers encountered were raised up in this instance. Hence, please allow us to retain this part. Thank you

In the revised version

Line #296-305

Amongst the barriers stated, common was community members' doubts about the effectiveness of deworming drugs, and their potential side effects [30], leading to their less involvement. Another barrier to CE was related to the communities' perceptions of the low quality of the government supplied drugs in the MDA compared to commercially available anthelminthics. For instance, a female participant in mass deworming stated, "*I feel like the drug (albendazole) does not automatically kill the worms because some would still come out alive. Unlike with Combantrin (the store-bought drug), worms passed with stool were actually dead*" [30]. Communities were finding it difficult to change previous practices of open defecation, according to a study on the use of low-cost sanitary latrines in Indonesia [29].

Q/

- Line 328: do you mean 8 studies?

Our reply/

The correct number of studies is ten for this revised version. Thank you.

In the revised version

Line # 310-311

The present review of ten studies from seven LMICs in the Asia Pacific Region revealed minimal/limited CE in the research.

Q/- Lines 332-335: the sentence is very long and unclear. Consider revising.

Our reply/

This section has been rephrased by being divided into two sentences. Thank you

In the revised version

Line # 314-317

The majority of the studies that composed this review were designed to report on health outcomes, with no description or evaluation of the level of community involvement in HSR. This revealed that rather than focusing on the level of CE in HSR, the primary objectives of these primary studies were to investigate health consequences.

Q/- Lines 340-341: the last sentence of the paragraph is unclear. Consider revising.

Our reply/

We have rephrased this part. Thank you

In the revised version

Line # 324-325

Similar to a published review [35], communities are regulated by power relations such as local governments, which participated in community-based interventions [25, 32].

Q/

- Discussion: the actual discussion seems to be missing – there is only a "summary of evidence", "study limitations" and "conclusions". A point to discuss is e.g. a comparison of the findings with the situation in other areas of public health, and/or other geographical areas. Other possible points to discuss include the optimal design of studies in this area, considerations under which circumstances community engagement is especially critical/not feasible, and others.

Our reply/

We very much appreciated these comments. We have already highlighted reviews that were published and mostly addressed about community involvement. This section has been updated for clarity. Thank you

In the revised version'

Line #325-330

According to a published review [15], community participation in PHC and water resource governance in South Africa was likewise tokenistic, regarding the Arnstein's ladder of involvement [36]. Since consultations and/or informing were the most typical forms of participation, the Arnstein's ladder of participation [36] was similarly appropriate to the degree of CE in our review of STH.

Line# 334-336

The main mechanisms of CE in this evaluation were small meetings with community leaders, community members, and health personnel, similar to prior reviews that evaluated CE [15,33,35, 36].

>>>>>>>>>>>>>>>>>>>>>>>>>>>

Reviewer #2: This study reports important information on CE during STH research that can aid in better planning and research on NTDS in the Asia Pacific.

Major remarks

Q/

Authors should refine/revise their search criteria; for example "hook worms" should be "hookworms". Further, some studies with clear CE seem to have not been included in their analyses. For example, in https://www.thelancet.com/journals/lanwpc/article/PIIS2666-6065(21)00167-X/fulltext and in https://www.mdpi.com/2414-6366/4/4/141

there is a clear CE, with head of the villages involved in the study. I would suggest author to re-perform their search. For example, the above study came up with a simple search of hookworm AND Asia AND community.

Our reply/

We very much appreciated your guidance and valuable inputs. We have updated search strategy by adding hookworms and trichuriasis. Following this, we have added two studies on the revised version. Thank you

In the revised version

Line# 132-135

The keywords with appropriate Bolen operators were used: “community engagement” “community participatory” “action research” “participatory research” “participatory action research” “community-based research” “soil transmitted helminths" " hook worms" " ascariasis" “ trichuriasis”

Line # 139

S1 Table

Q/

It is not clear why some exclusion criteria were chosen. For example, how some studies included in their analyses are different from those in table S2 is difficult to understand. Given the title of this manuscript, all research studies with a CE component in STH research should be included in the analyses. Some studies in S2 that were deemed as “efficacy” or “cross sectional” are nowhere different from those included in table 1.

Our reply/

In the inclusion criteria, we have described that programme/intervention in the study should involve community/stakeholder involvement or engagement and provide mechanisms and/or

processes of community engagement. Moreover, it should be targeted towards health services context. Thank you.

Minor remarks:

Q/

The text needs a thorough revision (e.g., often brackets are opened but not closed).

Our reply/

We have corrected these in the revised version. Thank you

Q/

Line 290: if out of 11, it shouldn't be that %

Our reply/

It is out of ten studies in the revised version. Hence, it becomes 70% (7/10). Thank you

In the revised version

Line # 219-220

Of them, ten studies across seven countries in the Asia Pacific region were eligible for this review [23-32].

Line # 277-278

Seven studies in this review (7/10, 70%) addressed the ranges of drivers that facilitated CE in HSR targeting STH [23, 25- 27-32].

Q/

Line 360: statement not needed

Our reply/

We have removed the statement from the revised version. Thank you

Q/

Line 368: missing a verb

Our reply/

We have corrected this sentence. Thank you

In the revised version’

Line# 357-358

Findings suggest that various forms of community had engaged in various aspects of the HSR context.
